# A lower bound on the number of mechanisms for discriminating fourth and higher order spatial correlations

**DOI:** 10.1186/1471-2202-16-S1-P154

**Published:** 2015-12-18

**Authors:** John WG Seamons, Marconi S Barbosa, Anton Bubna-Litic, Ted Maddess

**Affiliations:** 1 Eccles Institute for Neuroscience, John Curtin School of Medical Research, ANU, Canberra, ACT 0200, Australia

## 

The human visual system must employ mechanisms to minimize informational redundancy whilst maximizing dynamic range and maintaining that which is behaviorally relevant [1,2]. Previous research has concentrated on two-point correlation properties, as captured by spatial frequency and orientation tuning. There has been less research into higher-order correlations although they may inform us about cortical functioning [3]. Isotrigon textures can be used to probe the sensitivity of the human visual system. The obvious structure in isotrigons is exclusively due to 4^th ^and higher-order spatial correlations [4]. Thus, in order to discriminate isotrigons from noise, it is necessary to identify higher-order structure. Although artificially generated, the same structural features that give isotrigons salience also create salience in natural images [2].

Factor analysis can be used to infer the number of underlying independent neurological mechanisms which govern isotrigon discrimination. In this study, mean performance functions were calculated for two subjects using ten new isotrigons (VnL2) (Figure 1A). Two forms of factor analysis identified 3 principal factors (Figure 1B) [5]. Previous studies support that the number of mechanisms is less than 10 [6], and more likely 2-4 [7,8]. Such mechanisms may represent some combination of recursive or rectifying processes. Simple models of cortical processing, based on recursion, can discriminate isotrigons [9]. The formation of recursively applied products is physiologically plausible and can occur via dendritic back-propagation or dendritic spiking [10].

**Figure 1:**
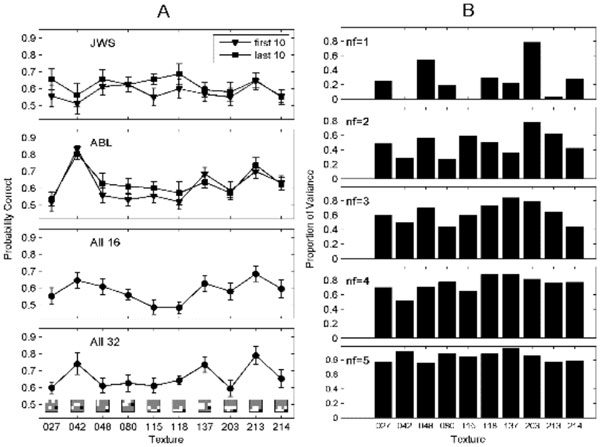
****A: **Mean performance functions for all subjects by texture type, presented separately according to subject and texture size**. For All16 (16x16 textures) and All32 (32x32) error bars are SE for n = 6 subjects. Glider shapes are shown in the bottom panel. **B: **Communalities for 5 different factor models. As the number of factors grows, the profile of bars becomes flatter indicating that the models progressively account for the data in a more balanced way. After nf = 3, the improvement in the reconstruction is marginal.

## References

[B1] Barlow H Redundancy reduction revisited Network 2001 12 3 241 253 11563528

[B2] Tkacik G Prentice JS Victor JD Balasubramanian V Local statistics in natural scenes predict the saliency of synthetic textures Proc Natl Acad Sci U S A 2010 107 42 18149 18154 20923876 10.1073/pnas.0914916107 PMC2964243

[B3] Victor JD Papathomas TVC, C.; Gorea, A; Kowler, E Isodipole Textures: A Window on Cortical Mechanisms of Form Processing Early Vision and Beyond 1995 MIT Press 99 107

[B4] Maddess T Nagai Y James AC Ankiewcz A Binary and ternary textures containing higher-order spatial correlations Vision Res 2004 44 11 1093 1113 15050814 10.1016/j.visres.2003.12.012

[B5] Seamons JWG Bubna-Litic A Barbosa MS Maddess T A Lower bound on the Number of Mechanisms for Discriminating Fourth and Higher Order Spatial Correlations Vision Res 2015 10.1016/j.visres.2014.12.023 25624152

[B6] Taylor RR Maddess T Nagai Y Spatial biases and computational constraints on the encoding of complex local image structure J Vis 2008 8 7 19 11 13 19146252 10.1167/8.7.19

[B7] Maddess T Nagai Y Discriminating isotrigon textures [corrected] Vision Res 2001 41 28 3837 3860 11738451 10.1016/s0042-6989(01)00226-7

[B8] Maddess T Nagai Y Victor JD Taylor RR Multilevel isotrigon textures J Opt Soc Am A Opt Image Sci Vis 2007 24 2 278 293 17206245 10.1364/josaa.24.000278

[B9] Nagai Y Taylor RR Loh YW Maddess T Discrimination of complex form by simple oscillator networks Network 2009 20 4 233 252 19919282 10.3109/09548980903373879

[B10] Hausser M Spruston N Stuart GJ Diversity and dynamics of dendritic signaling Science 2000 290 5492 739 744 11052929 10.1126/science.290.5492.739

